# Tailoring the Mechanical Properties of Al_0.4_CrFe_2_Ni_2_ Medium-Entropy Alloy via Thermomechanical Processing

**DOI:** 10.3390/ma19030502

**Published:** 2026-01-27

**Authors:** Róbert Kočiško, Patrik Petroušek, Ondrej Milkovič, Pavel Diko, Vladimír Girman, Andrea Sütőová, Michal Duchek, Michal Zemko

**Affiliations:** 1 Faculty of Materials, Metallurgy and Recycling, Technical University of Košice, Letná 9, 042 00 Kosice, Slovakia; patrik.petrousek@tuke.sk; 2 Institute of Materials Research, Slovak Academy of Sciences, Watsonova 47, 040 01 Košice, Slovakia; omilkovic@saske.sk (O.M.); vladimir.girman@upjs.sk (V.G.); 3 Institute of Experimental Physics, Slovak Academy of Sciences, Watsonova 47, 040 01 Košice, Slovakia; dikos@saske.sk; 4 Institute of Physics, Faculty of Science, P. J. Šafárik University in Košice, Park Angelinum 9, 041 54 Košice, Slovakia; 5 Department of Quality Management, VSB—Technical University of Ostrava, 17. Listopadu, 708 00 Ostrava, Czech Republic; andrea.sutoova@vsb.cz; 6 COMTES FHT a.s., Průmyslová 995, 334 41 Dobřany, Czech Republic; michal.duchek@comtesfht.cz (M.D.); michal.zemko@comtesfht.cz (M.Z.)

**Keywords:** medium-entropy alloys, thermomechanical processing, cryo-rolled, precipitation strengthening, microstructural evolution, mechanical properties

## Abstract

The microstructure and properties of a cobalt-free, cost-effective Al_0.4_CrFe_2_Ni_2_ medium-entropy alloy (MEA) after multi-stage thermomechanical processing, including annealing, rolling over a wide temperature range from hot to cryogenic conditions, and subsequent precipitation strengthening, were investigated in the present study. The initially cast microstructure was effectively homogenized through hot rolling with an 80% thickness reduction followed by homogenization annealing, resulting in the formation of a single-phase supersaturated solid solution and enhanced stability of plastic deformation. Strengthening of the MEA was achieved by rolling under both ambient and cryogenic conditions, with the deformation process predominantly governed by shear band formation. However, rolling under cryogenic conditions led to a more pronounced localization of plastic deformation, promoting the formation of deformation nanotwins and resulting in significantly higher strengthening compared to ambient rolling, with the alloy reaching a yield strength of 1040 MPa and an ultimate tensile strength of 1235 MPa. Precipitation hardening was governed by the formation of B2-type (ordered body-centered cubic, BCC) precipitates, which preferentially nucleated along deformation bands, thereby effectively strengthening the alloy to a yield strength of 1420 MPa and an ultimate tensile strength of 1465 MPa. Our results demonstrate that the investigated MEA offers a wide range of tunable mechanical properties, which can be effectively tailored through appropriate combinations of thermomechanical processing routes.

## 1. Introduction

High-entropy and medium-entropy alloys (HEAs and MEAs), based on approximately equiatomic proportions of multiple elements, represent a new generation of metallic materials that often exhibit mechanical properties exceeding those of conventional alloys. Many of these alloys are characterized, in particular, by high specific strength, a favorable combination of strength and ductility, excellent fracture toughness at low temperatures, outstanding fatigue resistance, and good creep resistance [1,2,3,4,5].

The design space of these alloys is very broad. In the field of structural materials, several approaches have been developed to enhance the synergy between high strength and good plasticity. One effective strategy is the formation of a multiphase microstructure, in which a ductile face-centered cubic (FCC) matrix is combined with a strengthening BCC phase. The formation of the strengthening BCC phase in HEA/MEA-type alloys is most commonly achieved by alloying with aluminum, which effectively shifts the phase stability from a single-phase FCC structure through a dual-phase region toward a BCC microstructure, as demonstrated for the Al_x_CoCrFeNi alloy system [6].

Although the presence of a BCC phase in the as-cast condition contributes to the strengthening of HEAs, its strengthening potential is not fully exploited. A significant increase in strength can be achieved through targeted microstructural modification via thermomechanical processing (TMP), which combines plastic deformation, annealing, and controlled phase transformations.

The Al_0.3_CoCrFeNi alloy is among the most extensively studied systems with respect to thermomechanical processing. It has been shown that after heavy cold rolling (60–90% thickness reduction), the microstructure can be further tailored by three different heat-treatment routes. Low-temperature annealing at approximately 600–700 °C leads directly to the formation of B2 and σ precipitates, resulting in high strength (YS ≈ 820 MPa, UTS ≈ 1050 MPa) but at the expense of reduced total elongation to fracture TE ≈ 35% [7,8]. In contrast, high-temperature recrystallization annealing produces a fine-grained structure as B2 precipitates effectively inhibit grain growth; the resulting microstructure exhibits lower strength (YS ≈ 400 MPa, UTS ≈ 800 MPa) but higher TE ≈ 45% [9,10,11,12]. By combining short-duration high-temperature recrystallization with subsequent precipitation annealing, a microstructure containing both B2 and L1_2_ phases can be obtained, providing a balanced combination of mechanical properties (YS ≈ 480 MPa, UTS ≈ 820 MPa, TE ≈ 45%) [7]. In conventional thermomechanical processing, cold rolling is most commonly employed; however, alternative approaches are increasingly being explored, including severe plastic deformation (SPD) techniques [13,14], hot rolling [15,16,17], and also rolling under cryogenic conditions [18], which strongly affect microstructural evolution and mechanical properties. At extremely low temperatures, recovery processes are suppressed, leading to a pronounced increase in dislocation density. Cryogenic deformation temperatures reduce the stacking fault energy (SFE) [19] and simultaneously activate deformation twinning mechanisms [20] or twining-induced plasticity (TWIP) and transformation-induced plasticity (TRIP) [21,22,23], thereby enhancing strength without a significant loss of ductility. It has also been demonstrated that alloying with aluminum reduces the stacking fault energy, which in turn promotes the activation of deformation twinning [24,25]. For the CoCrFeNiMn alloy [26], twinning mechanisms were shown to be more intense at 77 K than at 293 K, which was reflected in the tensile strength, increasing from UTS_293_ ≈ 1200 MPa to UTS_77_ ≈ 1500 MPa.

Although the Al-alloyed HEAs discussed above exhibit an attractive combination of mechanical properties achievable through appropriately designed thermomechanical processing, their practical applicability remains limited by the presence of expensive alloying elements, particularly cobalt. In response to this challenge, recent research has focused on Co-free or Co-lean alloy systems, and several compositions have already been partially explored, such as Fe_55_Mn_20_Cr_15_Ni_10_, AlCrFe_2_Ni_2_, Al_5_Fe_25_Cr_25_Ni_42.5_Ti_2.5_, and Al_5_Ti_2.5_Fe_25_Cr_25_Ni_42.5_ [18,26,27,28,29,30], in which the thermomechanical processing was predominantly based on cryogenic deformation routes.

The present work builds upon our previous study, in which various compositions of Al_x_CrFe_2_Ni_2_-type alloys were designed and evaluated [31]. Based on these findings, the current research focuses on enhancing the mechanical performance of the Al_0.4_CrFe_2_Ni_2_ alloy (where the subscripts denote atomic ratios), which represents a recently developed cobalt-free medium-entropy alloy concept that has not yet been systematically explored in terms of thermomechanical processing and precipitation hardening. The study compares deformation at room temperature with cryogenic rolling and analyzes the subsequent precipitation hardening behavior and thermal stability of this MEA alloy over a range of temperatures and holding times. Our results demonstrate that thermomechanical processing incorporating cryogenic rolling provides higher strength levels in the MEA alloy compared to conventional cold rolling.

## 2. Materials and Methods

### 2.1. Preparation of Experimental Material

The experimental material with the nominal composition Al_0.4_CrFe_2_Ni_2_ was prepared using vacuum induction melting. The induction melting was carried out under a vacuum of 10^−3^ Pa. Prior to melting, the furnace was evacuated and backfilled with high-purity argon gas twice. To ensure chemical homogeneity, the molten alloy was stirred by electromagnetic convection for 20 min at a temperature of 1600 °C. The melt was subsequently cast into a cylindrical Al_2_O_3_ mold with a diameter of 50 mm and a height of 120 mm, where it solidified inside the furnace under slow cooling conditions. High-purity elemental feedstock (Fe, Ni, Cr, Al) with a purity of ≥ 99.9 wt.% was used for alloy preparation.

### 2.2. Thermomechanical Processing

**Figure 1:**
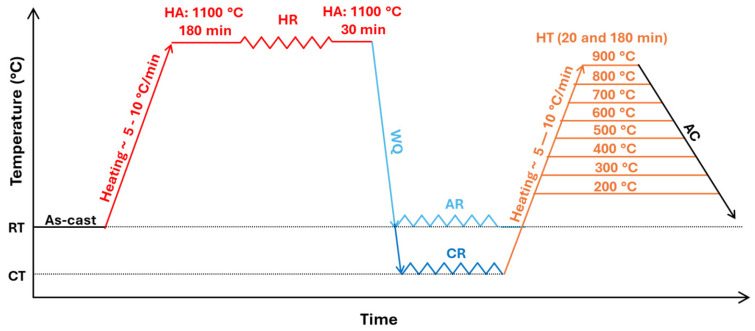
Thermomechanical processing schemes for the four rolling conditions investigated.

The thermomechanical processing routes applied to the investigated alloy are schematically illustrated in Figure 1. The processing was carried out in three main stages.

The first stage was aimed at homogenization of the as-cast microstructure and consisted of homogenization annealing (HA) at 1100 °C for 180 min. This was followed by hot rolling (HR), which was performed on an experimental DUO 550 two-high rolling mill equipped with flat cylindrical rolls at a rolling speed of 0.4 m·s^−1^. The cylindrical specimen with an initial diameter of 50 mm was hot rolled in nine passes to a final thickness of 7.8 mm, corresponding to a total thickness reduction (ε) of 80%. During hot rolling, the sample was reheated between individual passes to maintain a sufficiently high temperature and to avoid excessive strain localization or surface cracking. After hot rolling, the specimen reached a final width of approximately 73 mm and a length of about 435 mm, reflecting substantial material flow associated with the flattening of the initial cylindrical geometry. Immediately after deformation, the sample was re-homogenized at 1110 °C for 30 min and subsequently quenched to room temperature in a water bath.

The second processing stage focused on dislocation strengthening of the alloy through plastic deformation achieved by rolling at room temperature (AR) and under cryogenic conditions (CR), both with an ε = 60%. Rolling was carried out on an experimental DUO 180 two-high rolling mill equipped with flat cylindrical rolls at a rolling speed of 0.03 m·s^−1^. Cryogenic rolling was performed by first cooling the samples in liquid nitrogen for 20 min, followed by multi-pass rolling. To evaluate the evolution of mechanical properties for both rolling conditions, thickness reductions of ε = 20%, 40%, and 60% were analyzed.

The third processing stage was dedicated to precipitation hardening of the alloy in order to identify a synergistic balance between strength and ductility through heat treatment (HT). The heat-treatment temperatures and holding times were designed to achieve maximum precipitation strengthening while partially restoring ductility. Heat treatments were conducted at temperatures of 200, 300, 400, 500, 600, 700, 800, and 900 °C, with holding times of 20 min and 180 min, followed by free air cooling. Mechanical property measurements were performed only for selected processing conditions.

### 2.3. Microstructure Characterization

Phase analysis of the alloys was characterized using X-ray diffraction (XRD) performed on a Rigaku Rapid II X-ray diffractometer (Rigaku Holdings Corporation, Tokyo, Japan) equipped with a D-Max two-dimensional curved detector. Measurements were carried out in reflection geometry, with the omega angle fixed at 15°, while the phi axis was oscillated at a rate of 15° per second.

Microstructural analysis was conducted on samples prepared using standard metallographic procedures with Struers equipment, including sectioning, mounting, grinding, polishing, and etching. To reveal crystallographic features, as-cast samples were etched for 1–5 s in Marble’s reagent composed of HCl, CuSO_4_, and methanol, whereas rolled samples were etched using Glyceregia consisting of HCl, HNO_3_, and glycerol. The microstructure was examined using a Zeiss Axiovert A1 optical microscope (Carl Zeiss AG, Oberkochen, Germany).

For electron backscatter diffraction (EBSD) measurements, sample surfaces were subjected to final preparation by oxide polishing. Detailed microstructural observations were examined using a TESCAN MIRA 3 LMU scanning electron microscope (SEM; TESCAN, Brno, Czech Republic), with contrast enhanced through combined SE and BSE imaging. Energy-dispersive spectroscopy (EDS) was employed to determine the chemical composition of individual structural components, utilizing the Oxford Instruments X-act detector (Oxford Instruments, Abingdon, UK). Each analysis was performed in at least three separate regions per sample. The EBSD, using an Oxford Nordlys Max2 detector (Oxford Instruments, Abingdon, UK), was used to acquire phase maps of selected microstructures.

Observations using transmission electron microscopy (TEM) were performed with a JEOL 2100F UHR microscope (JEOL, Tokyo, Japan) equipped with a Schottky field-emission source and operated at 200 kV. Images were acquired in scanning transmission electron microscopy (STEM) and high-resolution modes, and selected-area electron diffraction (SAED) was used to identify the crystal structure. For TEM observations, the samples were prepared in the form of 3 mm discs that were first mechanically grounded and polished to a thickness of approximately 60 μm. These discs were subsequently thinned to electron-transparent foils by twin-jet electropolishing in a solution of 95% ethanol and 5% perchloric acid at −25 °C under a direct voltage of 18 V (Tenupol-5, Struers, Ballerup, Denmark). Ring SAED patterns were radially integrated employing software package Fit2D [32].

Thermal behavior of the experimental material was investigated using simultaneous thermal analysis (STA) based on the differential scanning calorimeter (DSC) method. The measurements were carried out with a STA-449-F1 Jupiter system (NETZSCH, Selb, Germany) at a heating rate of 10 K/min up to 950 °C. The analysis was conducted on as-cast samples weighing approximately 30 mg, placed in corund crucibles and heated under a high-purity nitrogen atmosphere.

### 2.4. Mechanical Testing

Static tensile tests were conducted for all investigated material conditions, including the as-cast state, the HR, AR, CR, and the HT. All tests were performed at ambient temperature in accordance with the EN ISO 6892-1 standard [33] using a Tinius Olsen H300KU universal testing machine (Tinius Olsen, Salfords, UK). A constant strain rate of 0.00025 s^−1^ was applied for all measurements. The TE was measured directly on the gauge length L0 of the specimen using a video extensometer. For each condition, at least three tensile specimens were tested. Based on the obtained stress–strain curves, the yield strength (YS), ultimate tensile strength (UTS), and elongation to fracture (A) were determined. Microhardness measurements were conducted using the Vickers method on Struers Duramin-5 hardness testers (Struers A/S, Ballerup, Denmark) in accordance with ISO 6507 [34]. For each evaluated condition, at least 15 indentations were made under a load of 981 mN (HV0.1) with a dwell time of 10 s. In the rolled conditions, the measurements were performed across the full thickness of the sample in order to minimize the effect of deformation heterogeneity introduced during rolling. From the measured values, the average microhardness and standard deviation were calculated for each condition.

## 3. Results

### 3.1. Microstructural and Mechanical Properties During Thermomechanical Processing

#### 3.1.1. Initial Alloy Microstructure

**Figure 2:**
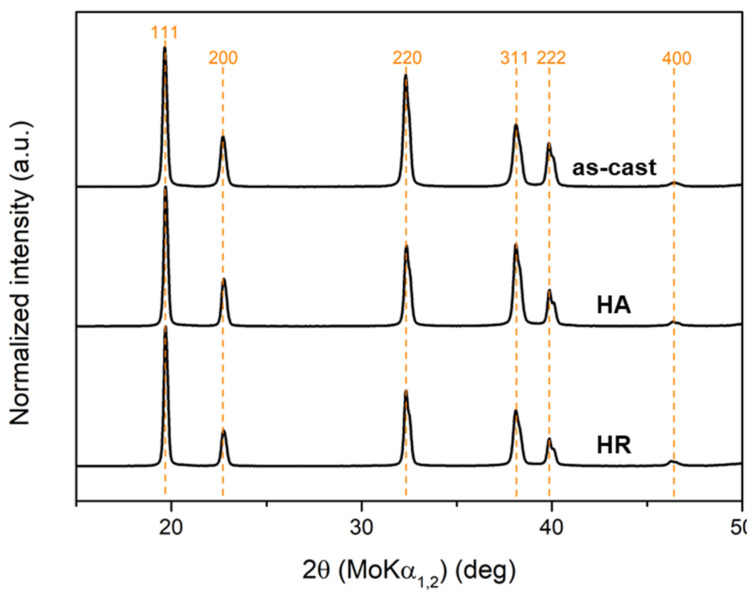
XRD patterns of the Al_0.4_CrFe_2_Ni_2_ alloy in the as-cast, homogenized, and hot-rolled states.

Figure 2 presents the XRD patterns of the Al_0.4_CrFe_2_Ni_2_ alloy in the as-cast, homogenized, and hot-rolled states. The results indicate that the alloy crystallizes exclusively in the FCC structure across all investigated conditions. The diffraction profiles exhibited well-defined peaks corresponding to the FCC phase, with reflections located at approximately 19° for the (111) plane, 22.5° for (200), 32.5° for (220), 37.5° for (311), and a weaker peak near 46° corresponding to (400). Based on the peak positions, the calculated lattice parameter of the FCC phase was a = 3.595 Å, which is consistent with reported values for similar Al-containing Cr-Fe-Ni alloys [26,35].

**Figure 3:**
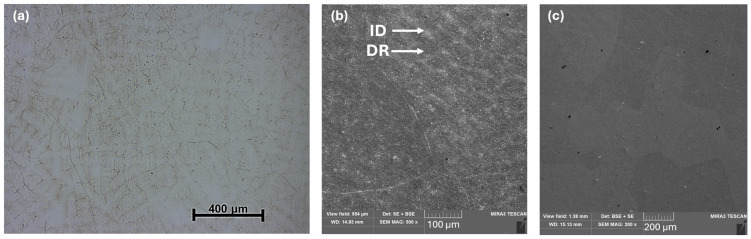
Microstructure of the Al_0.4_CrFe_2_Ni_2_ alloy: (**a**) as-cast, low-magnification optical micrographs; (**b**) as-cast, high-magnification SEM micrographs; and (**c**) microstructure after homogenization annealing.

Figure 3 shows the microstructures of the as-cast Al_0.4_CrFe_2_Ni_2_ alloy, which consists of large polyhedral grains with an average diameter of d ≈ 400 µm (Figure 3a). In addition, slight porosity was observed within the structure. Within these grains, there was local mixing of elements that were not yet bound by matrix interfaces, likely representing the dendritic (DR) nuclei that had formed, as can be seen in detail in Figure 3b. The total volume fraction of this phase was below 3%, which is why it was not detected by the XRD measurements. This type of structure was also observed in [6] as well as in our previous study [31], where the alloy was prepared by arc melting. The chemical composition of the DR (dark regions in the SEM image) was close to the nominal composition of the designed alloys, whereas the ID regions (bright regions in the SEM image) exhibited an increased content of Al and Cr. The chemical compositions of the individual structural phases and regions are presented in Table 1. After homogenization annealing at 1100 °C for 3 h, the alloy exhibited a homogeneous single-phase microstructure in which the average size of the polyhedral grains increased slightly to approximately 300–400 µm (Figure 3c). The individual grains displayed varying crystallographic orientations.

#### 3.1.2. Microstructure Evolution During Hot Rolling

**Figure 4:**
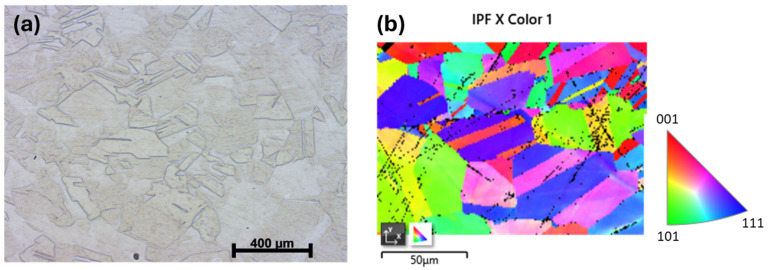
Microstructure of the Al_0.4_CrFe_2_Ni_2_ alloy after hot rolling (HR). (**a**) Optical Micrograph, (**b**) IPF map obtained from EBSD analysis.

Figure 4 shows the microstructure obtained after hot rolling, followed by homogenization at 1100 °C for 30 min and subsequent water quenching. This microstructure (Figure 4a) exhibited a recrystallized polyhedral grain structure with grain size in the range of 50–100 µm, accompanied by a relatively high fraction of annealing twins, with no porosity observed after hot rolling. The inverse pole figure (IPF) map obtained from EBSD analysis (Figure 4b) revealed a polycrystalline microstructure with a wide range of crystallographic orientations. Annealing twins, characteristic of FCC HEA alloys, were observed within the equiaxed recrystallized grains and appeared as sharp orientation changes between adjacent regions.

#### 3.1.3. Microstructural Evolution During Rolling at Ambient Temperature Rolling

**Figure 5:**
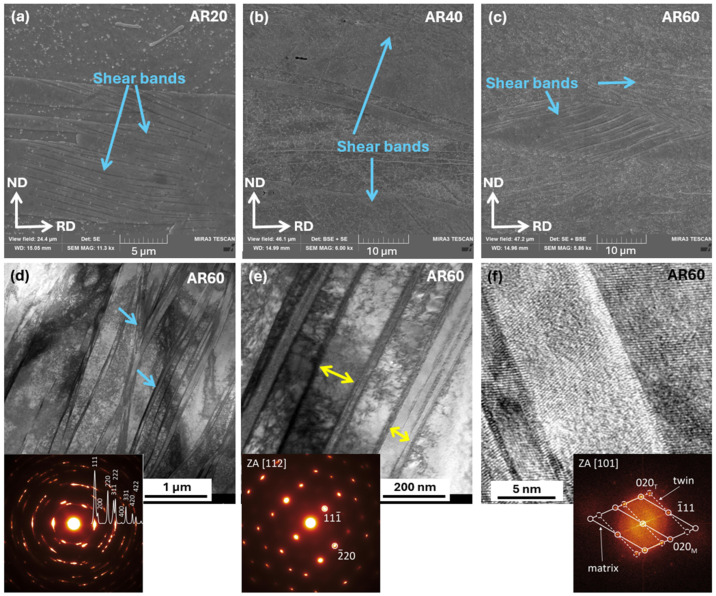
SEM and TEM microstructures of the alloy after ambient rolling to thickness reductions of (**a**) 20%, (**b**) 40%, and (**c**) 60%. (**d**,**e**) STEM bright-field (BF) microstructural features, together with the corresponding SAED patterns. (**f**) HRTEM nanoscale details of the shear bands, with the corresponding FFT patterns confirming the FCC structure.

Figure 5 illustrates the evolution of the microstructure during plastic deformation induced by rolling at an AR under thickness reductions of 20%, 40%, and 60%. Throughout the deformation process, the alloy exhibited pronounced deformation heterogeneity, which is primarily manifested by the formation of shear bands. At a 20% thickness reduction (AR20), shear bands were observed only in a limited number of grains (Figure 5a), appearing as narrow, highly deformed regions with an increased density of slip lines, which indicates strong localization of plastic deformation. With increasing deformation to 40% (AR40), a pronounced increase in the density and spatial extent of shear bands was observed (Figure 5b). Upon further increasing the thickness reduction to 60% (AR60), shear bands became the dominant microstructural feature, extending across entire grains or groups of grains and forming continuous networks throughout the material volume (Figure 5c). Further insight into the nature of the shear bands in the 60% deformed state was provided by TEM analysis, which, together with the corresponding SAED pattern, indicated a severely deformed microstructure exhibiting pronounced deformation texture. The TEM observations in Figure 5d,e revealed nanoscale shear bands with thicknesses of approximately 10–50 nm (marked by blue arrows), forming dense lamellar deformation zones within individual grains, while the spacing between adjacent bands ranged from about 90 to 150 nm (marked by yellow arrows). The regions between the shear bands exhibited a heavily deformed microstructure characterized by a high density of dislocations. The SAED diffraction pattern acquired from the region between the shear bands (Figure 5e) exhibited regular diffraction spots corresponding to a single crystallographic phase throughout the analyzed volume. In some regions within the shear bands, boundaries corresponding to deformation nanotwins were identified, as documented by the High Resolution Transmission Electron Microscopy (HRTEM) image in Figure 5f and analyzed from the Fast Fourier Transformation (FFT) pattern [36]. The FFT pattern showed pronounced symmetry and paired splitting of diffraction spots, which is characteristic of deformation twins in an FCC lattice, mirror-related with respect to the (111) plane.

#### 3.1.4. Microstructural Evolution During Cryogenic Rolling

**Figure 6:**
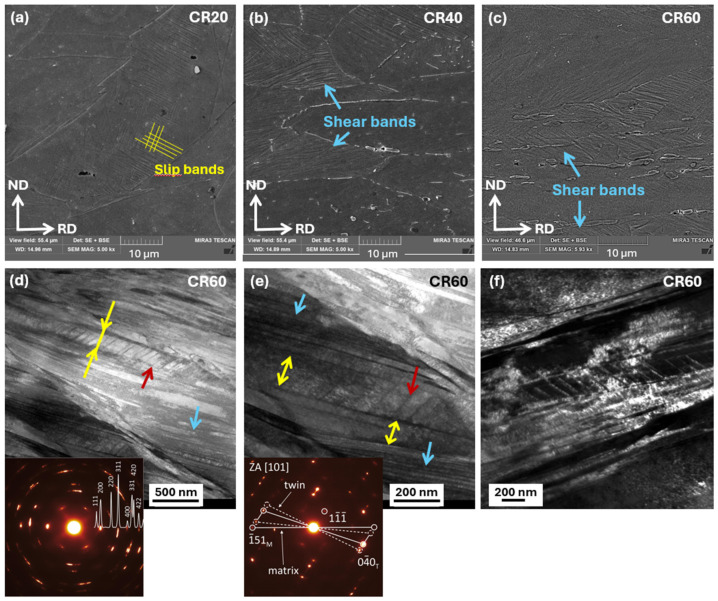
SEM and TEM microstructures of the alloy after ambient rolling to thickness reductions of (**a**) 20%, (**b**) 40%, and (**c**) 60%. (**d**) STEM bright-field (BF) detailed microstructural features, together with the corresponding SAED patterns confirming the presence of a single phase, (**e**) STEM bright-field (BF) detailed microstructural features, together with the corresponding SAED patterns confirming the presence of twins, and (**f**) TEM dark-field (DF) images highlighting twins.

Figure 6 presents the microstructural evolution induced by cryo-rolling at thickness-reduction levels of 20%, 40%, and 60%. The microstructural development at the initial deformation stage (20%, Figure 6a) differed from that observed during room-temperature rolling, as the deformation is governed by the slip-band mechanism. This mechanism manifests as localized slip of dislocations along one or several intersecting slip planes within individual grains. As the deformation increases under cryogenic conditions (40%), the dominant deformation mechanism shifts from slip bands to shear bands. The grains become more elongated in the rolling direction, and a larger portion of them is deformed by shear bands. At a deformation level of 60% (CR60), a substantial portion of the microstructure was affected by shear bands, which became the dominant deformation mechanism, as clearly demonstrated in the STEM images shown in Figure 6d,e. Between these bands, regions containing very fine lamellae with a thickness of approximately 25 nm (highlighted in blue) can be distinctly observed, as well as bands containing nanotwins that are oriented transverse to the shear bands and form narrow lamellar features interrupting the otherwise continuous shear-band structure (highlighted in red). Their presence and orientation are clearly documented in the STEM bright-field detail shown in Figure 6e, which are further supported by the corresponding diffraction pattern. Their presence was also confirmed by the dark-field TEM image in Figure 6f, where the nanotwin lamellae appeared sharply delineated against the surrounding heavily deformed matrix. A high density of dislocations can be observed in the microstructure.

### 3.2. Mechanical Properties After Homogenization of the As-Cast State and After Alloy Strengthening by Rolling Under Ambient and Cryogenic Conditions

**Figure 7:**
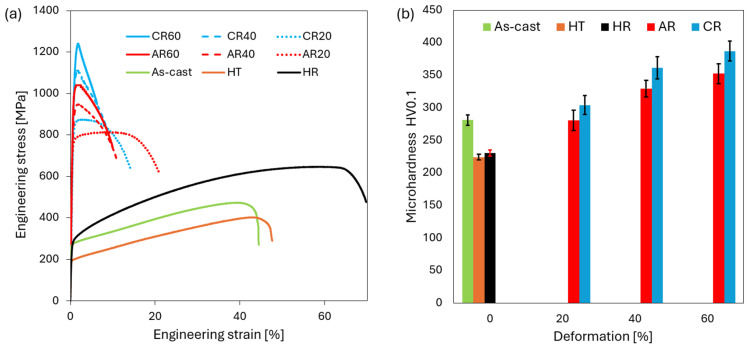
Mechanical properties of the as-cast, homogenized, hot-rolled, ambient-rolled, and cryogenically rolled conditions: (**a**) engineering tensile stress–strain curves; (**b**) microhardness (HV0.1).

The mechanical properties of the alloy in the as-cast, homogenized, hot-rolled, ambient-rolled, and cryo-rolled conditions are summarized in Figure 7, while the corresponding numerical values are listed in Table 2. In the initial as-cast condition, the material exhibited a relatively low yield strength (YS = 268 MPa) and ultimate tensile strength (UTS = 474 MPa), but a comparatively good total elongation to fracture (TE up to 44.9%) considering its cast microstructure. Homogenization annealing resulted in a decrease in YS, UTS, and hardness (from 281 HV0.1 to 224 HV0.1), accompanied by a slight increase in total elongation to fracture and reduction in area, reaching TE = 48.5% and RA = 59.9%. A more effective improvement in mechanical properties was achieved through hot rolling, where intensive deformation with an 80% thickness reduction, together with ongoing recrystallization processes, significantly enhanced the material’s performance. This improvement was most evident in the increased total elongation to fracture and reduction in area (70% and 69%, respectively), indicating markedly enhanced formability, reflected also in a UTS/YS ratio of 2.2. This reserve of uniform deformation was slightly higher than that of austenitic stainless steel AISI 316L and was comparable to that reported for the HEA and MEA alloys [37].

Subsequent rolling under ambient conditions led to a gradual increase in strength and hardness with increasing deformation. At a 20% thickness reduction, the yield strength reached 738 MPa and the ultimate tensile strength 807 MPa, while the total elongation to fracture decreased to 21%, with a uniform elongation of about 13%. An almost complete loss of uniform elongation (≤2%) was observed at thickness reductions of 40% and 60%. The highest degree of strain hardening was achieved at 60% deformation, where the yield strength increased to 839 MPa and the ultimate tensile strength to 1045 MPa, accompanied by a reduction in total elongation to fracture to approximately 10%. Such a mechanical response is characteristic of materials with an ultrafine microstructure, in which the high density of grain boundaries and defect interfaces promotes intensive strain hardening but simultaneously limits the material’s ability to sustain uniform plastic deformation. As a result, these materials typically exhibit high strength combined with a pronounced reduction in ductility as deformation progresses. Rolling under cryogenic conditions results in a more pronounced strengthening effect compared to ambient-temperature rolling, which is attributed to the higher fraction of shear bands observed in the microstructure that promote intensive dislocation accumulation and suppress dynamic recovery. After 40% deformation, the material exhibited higher strength levels (YS = 962 MPa and UTS = 1105 MPa) than those achieved after 60% ambient-temperature rolling, indicating that the cryogenic deformation route was more effective in promoting strain hardening despite the lower overall thickness reduction. After a 60% thickness reduction during the cryo-rolling (CR) process, the highest tensile strength of 1235 MPa and a hardness of 387 HV0.1 were achieved, which is comparable to the CoCrFeNi [20] HEA processed under the same deformation conditions. This behavior can be attributed to the high density of shear bands combined with the formation of nanotwins between them, both of which act as effective barriers to dislocation motion and thus significantly enhance the material’s strength.

### 3.3. Influence of Post-Rolling Annealing on Microstructural Development and Mechanical Performance

**Figure 8:**
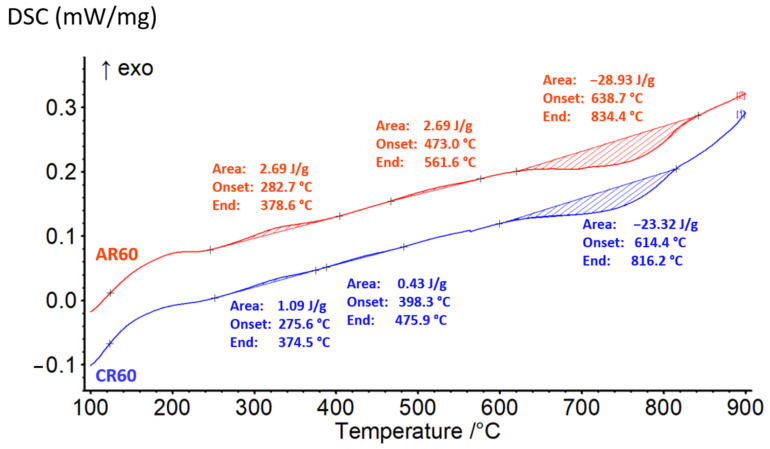
DSC heat–flow curves of the AR60 and CR60 conditions recorded from room temperature to 900 °C.

The alloys exhibiting the highest strengthening, processed under ambient and cryogenic rolling conditions (AR60 and CR60), were further subjected to heat treatment according to the scheme shown in Figure 1 to evaluate the extent of precipitation strengthening and determine the thermal stability of their mechanical properties. At each annealing temperature and holding time, the evolution of the mechanical properties was monitored by measuring hardness. To identify the thermally activated processes occurring in the investigated alloy within the temperature interval from room temperature to 900 °C, differential scanning calorimetry (DSC) was employed. In both processing conditions (AR60 and CR60), the alloy revealed two exothermic peaks followed by a single endothermic peak, as illustrated in Figure 8.

**Figure 9:**
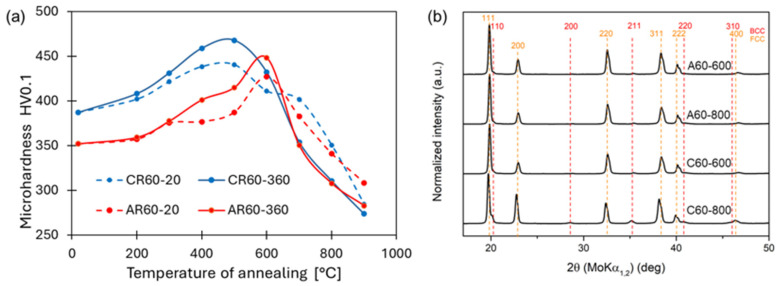
(**a**) Evolution of microhardness (HV0.1) of the AR60 and CR60 conditions as a function of annealing temperature (200–900 °C) for holding times of 20 min and 180 min; (**b**) XRD patterns of the AR60 and CR60 conditions annealed at 600 °C and 800 °C for 180 min.

The exothermic reaction observed in Al-containing HEA/MEA systems is most likely associated with the precipitation of ordered L1_2_ and B2-type particles [8,38] from the FCC matrix. The first exothermic peak appeared in nearly the same temperature range for both processing conditions, occurring between approximately 275 °C and 378 °C. This peak can be attributed to the formation of nanoscale precipitates, whose strengthening effect is clearly reflected in the corresponding increase in hardness from 352 HV0.1 to 401 HV0.1 for AR, and from 387 HV0.1 to 459 HV0.1 for CR, as shown in Figure 9b. At shorter annealing times, the strengthening effect is less pronounced; therefore, the following analysis will focus on samples annealed for longer durations.

**Figure 10:**
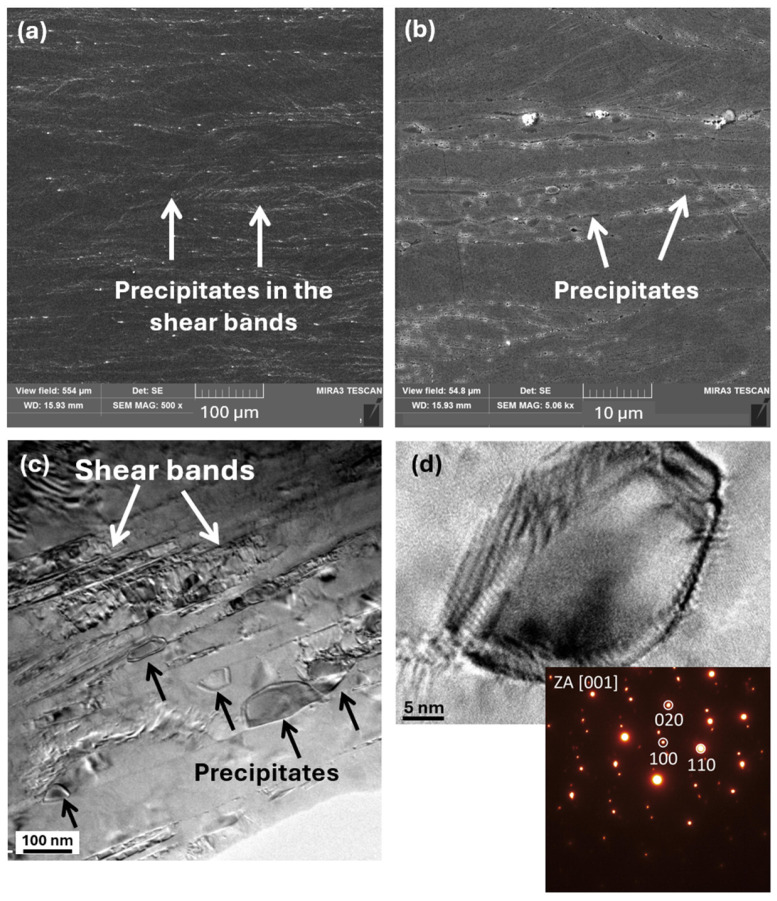
SEM and TEM microstructures of the AR60 condition annealed at 600 °C. (**a**,**b**) show SEM images at two different magnifications. (**c**,**d**) present TEM micrographs illustrating the distribution of precipitates, (**d**) providing a detailed HRTEM view of an individual precipitate, together with the corresponding SAED patterns confirming the presence of B2-type (BCC) precipitates.

The second exothermic peak shifted to lower temperatures in the CR-processed alloy, occurring between approximately 398 °C and 476 °C, whereas in the AR condition, it appeared at higher temperatures, in the range of 473 °C to 561 °C. This second exothermic reaction is also associated with the formation and growth of precipitates, accompanied by an increasing degree of atomic ordering, similar to what has been reported for HEA alloys with an FCC structure annealed at 500–550 °C [39,40]. The temperature shift observed for the CR condition can be attributed to the more intense deformation introduced during cryogenic rolling, which results in a higher level of stored internal energy. This elevated stored energy facilitates the onset of thermodynamic processes at lower temperatures compared to the ambient-rolled alloy. The maximum hardness for the AR60 condition was achieved at 600 °C for 180 min, where the alloy reached a hardness of 449 HV0.1. At this annealing temperature, the AR60-600 microstructure exhibited fine precipitates arranged in chain-like formations along the shear bands, as shown in Figure 10a,b. A similar type of precipitation has also been reported in the Al_0.3_CoCrFeNi alloy after AR and 4 h of annealing at 700 °C [8]. These precipitates, with sizes below 100 nm, form as a result of enhanced diffusion mobility and the local accumulation of deformation within the shear bands. They subsequently act as effective obstacles to dislocation motion, thereby contributing to their pinning effect and to the localized strengthening of the material. As shown in Figure 9b, the peaks of the BCC phase appeared in the XRD pattern after annealing at 600 °C with its dominant reflections identified at the (110) (near 20°), (200) (near 28°), (211) (near 35°), and (220) (near 41°) planes. The lattice parameters of the structural constituents for the AR60 and CR60 conditions annealed in the temperature range of 500–800 °C are summarized in Table 3. A bright-field TEM image of the AR60-600 condition is shown in Figure 10c, clearly revealing precipitates arranged along the shear-band lines. The provided SAED pattern (Figure 10d) corresponded to a BCC phase based on the arrangement of its reflections. The presence of forbidden reflections, such as (100), alongside strong allowed for reflections like (110) and (020), indicating a B2-ordered structure. The uneven radial distribution of reflection intensities further serves as a strong indicator of a highly ordered structure. The precipitates exhibited an ellipsoidal morphology with lengths of approximately 100–150 nm and widths of about 50 nm, and HRTEM analysis revealed that they possessed coherent interfaces with the surrounding matrix. Several studies have confirmed that in Al-alloyed MEA/HEA alloys, precipitation of the B2 phase with a BCC-type structure occurs [8,9,39,41,42].

**Figure 11:**
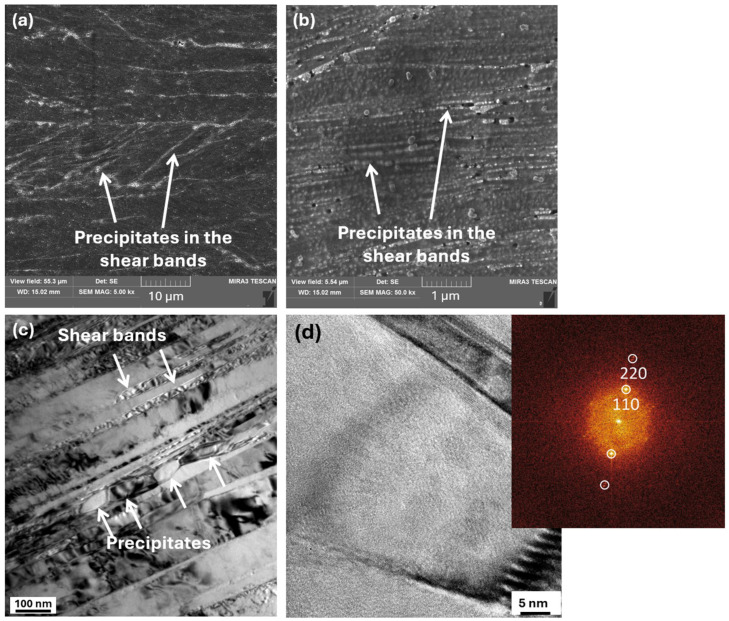
SEM and TEM microstructures of the CR60 condition annealed at 500 °C. (**a**,**b**) show SEM images at two different magnifications. (**c**,**d**) present TEM micrographs illustrating the distribution of precipitates, (**d**) providing a detailed HRTEM view of an individual precipitate, together with the corresponding FFT patterns confirming the presence of B2-type (BCC) precipitates.

The maximum hardness of the CR60 condition was recorded after annealing at 500 °C, reaching 468 HV0.1, which was 100 °C lower than the annealing temperature required to achieve peak hardness in the AR60 condition. Its microstructure exhibited a higher number of fine precipitates compared to AR60-600, likewise, arranged in chain-like formations along the shear bands, as shown in Figure 11a,b. The higher intensity of the BCC peaks in the XRD pattern (Figure 9b) further confirms the more pronounced precipitation of the secondary phase. Figure 11c shows a bright-field TEM image of the CR60-500 condition, revealing precipitates preferentially arranged along shear-band lines. A detailed HRTEM image of the precipitate together with its FFT pattern is shown in Figure 11d, indicating the presence of a B2-type (BCC) phase. The increased number of precipitates in the alloy rolled under cryogenic conditions, even at a lower annealing temperature, indicates that precipitation is strongly governed by the accumulated deformation energy, which reduces the activation energy required for precipitate nucleation and growth. This mechanism of deformation-induced precipitation has also been observed in the Al_0.5_CoCrFeNiCu alloy [43].

**Figure 12:**
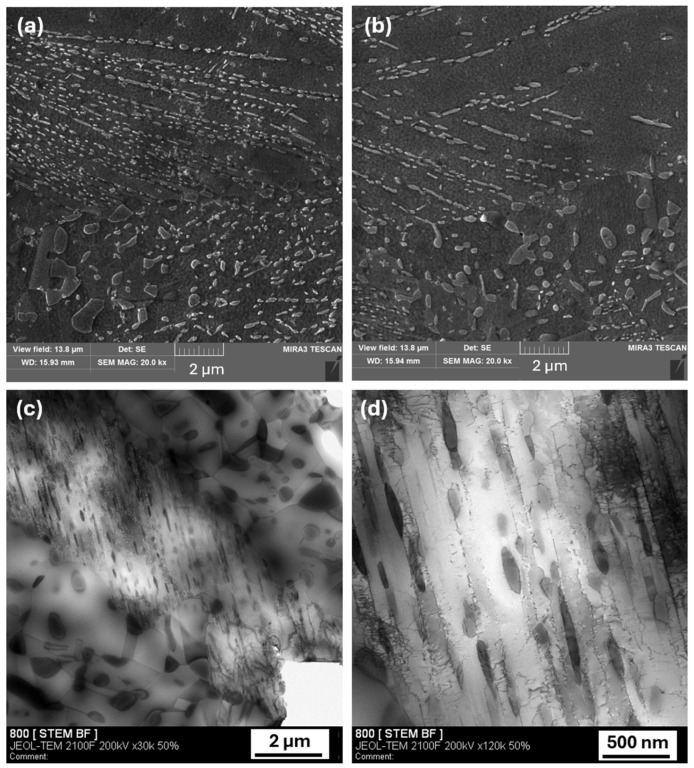
SEM and TEM microstructures after annealing at 800 °C: (**a**) SEM image of the AR60 condition; (**b**) SEM image of the CR60 condition; (**c**,**d**) detailed bright-field STEM micrographs of precipitate distributions, where (**c**) shows a partially recrystallized region with coarse precipitates and (**d**) a region containing fine precipitates.

A pronounced endothermic reaction on DSC curves was observed for both the AR and CR conditions within nearly the same temperature range, extending from approximately 614 °C to 834 °C. During annealing at 800 °C, both the AR60 and CR60 conditions exhibited small and large precipitates in their microstructures. The small precipitates, with sizes of 120–240 nm, contained 18.79 at.% Al and were aligned along the original shear-band direction. In contrast, the larger precipitates, ranging from 600 to 800 nm, formed within the recrystallized grains and exhibited a higher Al content of 27.63 at.% at the expense of Cr and Fe. Their chemical composition is provided in Table 4. At these elevated temperatures, enhanced atomic diffusion promotes several thermally activated processes typical of MEA and HEA systems. These include the coarsening of fine precipitates to sizes at which they no longer provide effective strengthening, as well as the simultaneous annihilation of dislocations. The combination of precipitate coarsening and dislocation recovery effectively eliminates both precipitation strengthening and dislocation strengthening. Consequently, these processes lead to a significant reduction in strength accompanied by an increase in ductility. Hardness measurements strongly support this interpretation. Under CR60-600 conditions, a decrease of approximately 10% is already observed at 600 °C. A much more pronounced reduction in hardness, by about 32%, occurs at 800 °C, indicating the onset of significant microstructural softening (308 HV0.1). All of these structural features can be identified in detail using STEM analysis of the CR60-800 condition, as shown in Figure 12c,d. The microstructure consisted of recrystallized regions containing large precipitates, as well as shear-band regions in which small oval precipitates were distributed along the band boundaries, as illustrated in Figure 12d. Within the shear bands, a reduction in dislocation density was also observed. With further increases in temperature, the hardness continued to decline steadily, reaching values as low as 280 HV0.1 at 900 °C, which reflects extensive recovery and recrystallization processes.

**Figure 13:**
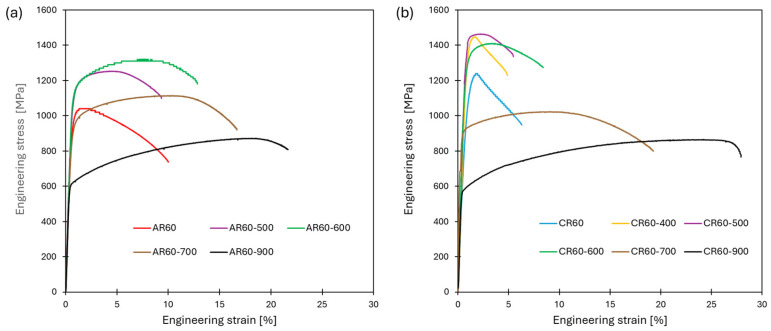
Engineering tensile stress–strain curves as a function of annealing temperature (500–900 °C) for holding times of 20 and 180 min for the (**a**) AR60 and (**b**) CR60 conditions.

Figure 13 shows selected engineering tensile stress–strain curves for the AR60 and CR60 conditions after heat treatment at temperatures ranging from 500 to 900 °C for 180 min, while the corresponding mechanical property values are summarized in Table 5. In both cases, the increase and subsequent decrease in mechanical properties were fully consistent with the evolution of the microstructure and hardness presented above. In the case of the AR60 condition (Figure 13a), the highest values of YS = 1130 MPa and UTS = 1320 MPa at a total elongation to fracture of 12.8% were achieved at an annealing temperature of 600 °C. It should be noted that during precipitation strengthening at 500 °C and 600 °C, both the uniform elongation and the total elongation to fracture gradually increased compared to the as-deformed state. With increasing annealing temperature up to 900 °C, a pronounced decrease in strength was observed alongside an increase in ductility, with the AR60-900 condition reaching YS = 613 MPa and UTS = 872 MPa at a TE = 21.7%.

For the CR60 condition (Figure 13b), the maximum strength levels were achieved after annealing at 500 °C, where the alloy reached YS = 1420 MPa and UTS = 1465 MPa, with a total elongation to fracture of 5.5%. At this stage, a slight increase in uniform elongation was observed at 400 °C and 500 °C, although the improvement was considerably less pronounced than in the AR60-600 condition. Once the annealing temperature exceeded 600 °C, the alloy began to exhibit a progressive decrease in strength, consistent with the hardness evolution discussed earlier. At the highest annealing temperature, the mechanical response approached that of the AR60-900 condition, attaining YS = 477 MPa and UTS = 859 MPa, accompanied by a markedly improved total elongation to fracture of 27.8%.

## 4. Discussion

### 4.1. Homogenization of the As-Cast Microstructure of Al_0.4_CrFe_2_Ni_2_

In the first stage of thermomechanical processing, the effect of hot deformation and homogenization annealing on the evolution of the microstructure and mechanical properties of the as-cast alloy was analyzed.

The initial coarse-grained as-cast microstructure, characterized by aluminum segregation in the interdendritic (ID) regions, was chemically homogenized after annealing at 1100 °C. Subsequent hot deformation to a thickness reduction of 80%, combined with homogenization annealing at 1100 °C for 30 min, resulted in the formation of a polyhedral microstructure with an average grain size of 50–100 μm and an increased fraction of annealing twins. This microstructure, typical of FCC materials with low stacking fault energy, provides favorable conditions for enhanced ductility and microstructural stability, which is particularly important for subsequent deformation processing at ambient temperatures [44]. Rapid cooling of the alloy from the annealing temperature after hot rolling ensured the formation of a single-phase supersaturated solid solution, which was also confirmed by XRD measurements.

**Figure 14:**
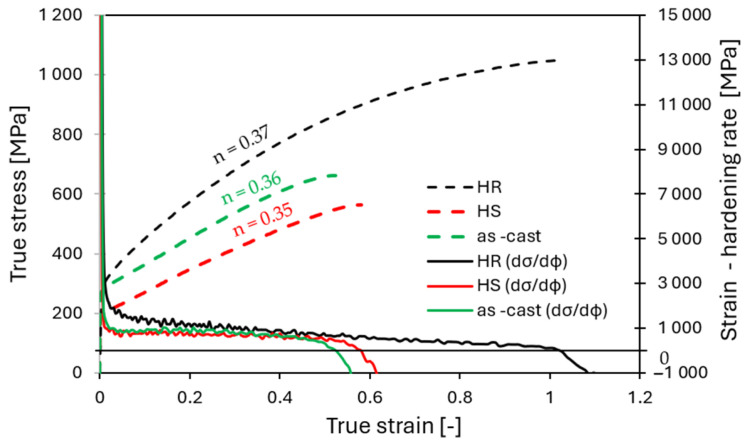
Tensile properties of the as-cast, HS and HR conditions. True stress–strain curves and corresponding work-hardening rate.

Figure 14 presents the true stress–strain curves and the corresponding strain hardening rates for the as-cast, HS, and HR conditions. A comparison of these curves revealed distinct differences in the deformation behavior depending on the microstructural state. The as-cast and HS conditions, characterized by coarse-grained microstructures with grain sizes exceeding 300 μm, exhibited a lower strain hardening exponent and a more rapid decrease in strain hardening capacity, indicating limited dislocation accumulation and an earlier onset of plastic deformation localization. In contrast, the HR condition with a refined microstructure exhibited a higher strain hardening exponent, suggesting more effective interactions between dislocations and grain boundaries and a more stable plastic deformation process. Refinement of the initial as-cast microstructure thus creates more favorable conditions for subsequent deformation processing at ambient temperatures. This trend is consistent with literature reports for various HEA systems, where thermomechanical processing has been shown to simultaneously enhance both strength and total elongation to fracture [15,16,17,45].

### 4.2. Strengthening of the Alloy by Plastic Deformation

Plastic deformation of the alloy at ambient temperature is primarily governed by the localization of plastic flow into shear bands, which represent the dominant mechanism of strain hardening. At higher deformation levels (60%), this mechanism is accompanied by the formation of deformation nanotwins aligned along the shear bands, which further contribute to the strengthening and stabilization of plastic deformation [36,46,47,48,49].

In contrast, during the initial stages of deformation under cryogenic conditions, a different deformation mechanism was observed compared to deformation at ambient temperature. At a thickness reduction of 20%, deformation was accompanied by the formation of slip bands, which arise due to the strong localization of dislocation motion along a limited number of slip planes. Such behavior is characteristic of materials deformed at low or cryogenic temperatures, where the reduced stacking fault energy suppresses dislocation cross-slip and promotes their accumulation in narrow bands, thereby facilitating the formation of slip bands. A similar mechanism was also observed in our previous study on AISI 316LN steel [50]. With increasing levels of cryogenic deformation, the deformation behavior gradually changed, and already at 40% thickness reduction, the dominant mechanism became the formation of shear bands. This mechanism persisted at 60% deformation but with significantly higher intensity, which was further enhanced by the presence of deformation nanotwins oriented transverse to the shear bands. These results suggest that as a consequence of strong localization of plastic deformation within shear bands, a pronounced accumulation of dislocations and an increase in local shear stress occur. Under these conditions, and additionally supported by the reduced stacking fault energy, deformation twinning becomes energetically more favorable than further dislocation slip. Similar behavior has been reported for several alloy systems [18,27,51].

**Figure 15:**
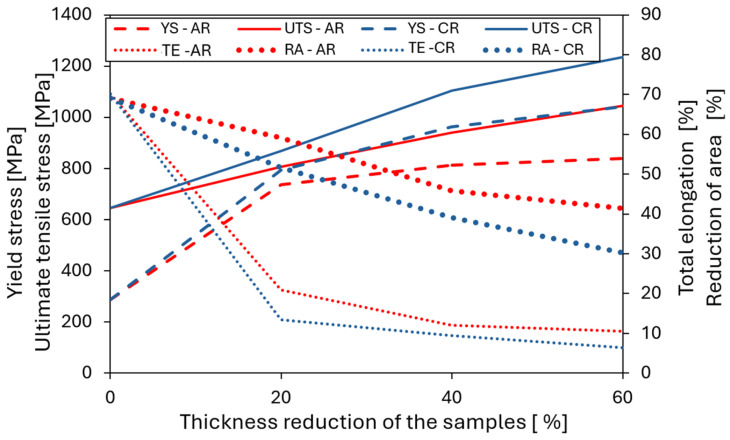
Evolution of mechanical properties as a function of thickness reduction in samples during AR and CR rolling.

The microstructural evolution is directly reflected in the mechanical properties shown in Figure 15. At low thickness reductions (~20%), the most pronounced increase in yield strength was observed, which was associated with rapid dislocation accumulation in the initially weakly deformed microstructure and with the onset of shear band formation during deformation at AR or slip band formation during CR. In contrast, at higher deformation levels (40% and 60%), the increase in yield strength became more moderate, indicating a gradual saturation of dislocation density and stronger localization of plastic deformation into shear bands. Nevertheless, the CR states achieved higher yield strength values due to a higher density of shear bands compared to the states deformed at AR [26]. At the same time, the CR states exhibited a more pronounced reduction in ductility and area reduction, particularly at higher deformation levels, reflecting a higher degree of plastic flow localization.

### 4.3. Precipitation Hardening of the Alloy and Its Thermal Stability

**Figure 16:**
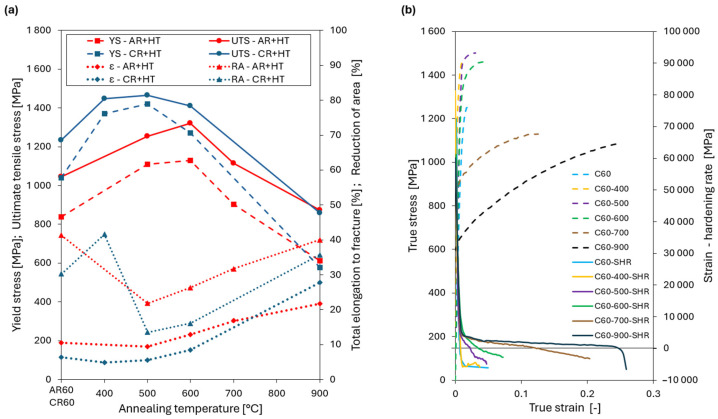
(**a**) Tensile properties of the thermomechanical treatment MHEAs. (**b**) True stress–strain curves and corresponding work-hardening rate for CR60 state.

Annealing of the investigated Al_0.4_CrFe_2_Ni_2_ alloy after severe plastic deformation is intended to further enhance its strength through precipitation hardening. The DSC analysis revealed the presence of both precipitation and recovery processes, with precipitation occurring in two distinct temperature ranges. In the peak-aged condition (AR60-600 and CR60-500), B2-type precipitates with a BCC crystal structure were identified. Their nucleation and uniform growth, preferentially within shear band regions, significantly contribute to alloy strengthening. This effect was manifested primarily by an increase in yield strength, which rose by approximately 300 MPa after ambient rolling and by about 400 MPa after cryogenic rolling, while the ultimate tensile strength increased by approximately 300 MPa (AR) and 230 MPa (CR) compared to the as-rolled condition, as shown in Figure 16a. The true stress–true strain and strain-hardening rate (SHR) versus true strain relationships (Figure 16b) reveal that annealing at 400 °C leads predominantly to precipitation strengthening of the alloy. In contrast, annealing at 500 °C indicates not only further precipitation hardening but also the onset of partial recovery in the microstructure, as evidenced by the reduced slope of the SHR curves.

The structural state after rolling also has a pronounced effect on the thermal stability of the alloy. In the AR60 condition, annealing at 500 and 600 °C resulted not only in a gradual increase in yield strength and ultimate tensile strength, but also in an increase in uniform elongation, with A_g,CR60−600_ reaching approximately 7% (Figure 13a). This evolution of mechanical properties indicates that precipitation strengthening is accompanied by partial relaxation of the dislocation substructure.

**Figure 17:**
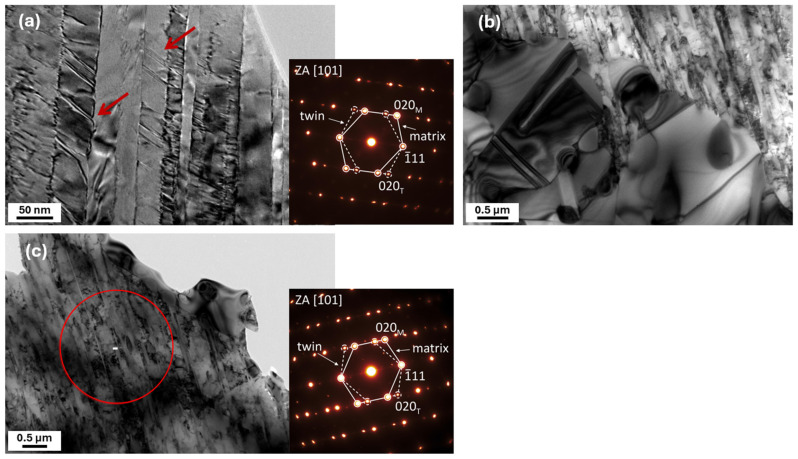
TEM microstructural evolution of the CR-processed alloy after annealing: (**a**) bright-field (BF) image and SAED pattern of the CR60 condition annealed at 500 °C; (**b**,**c**) bright-field (BF) images of the CR60 condition annealed at 800 °C, where (**b**) shows partial recrystallization and annealing twins and (**c**) reveals deformation nanotwins in shear-band regions.

At higher annealing temperatures in the range of 700–900 °C, a gradual loss of dislocation strengthening occurs. In contrast, the alloy processed by cryogenic rolling retains a pronounced dislocation-strengthening character up to temperatures of approximately 400–500 °C (Figure 17b). It should be noted that at 500 °C, a slight increase in uniform elongation was observed, which was also reflected in the strain–hardening rate curve (purple curve) in Figure 17b, where a subtle change in slope could be detected. Even at 600 °C, the alloy still preserved dislocation strengthening to a significant extent.

The enhanced thermal stability of the CR condition is closely associated with the presence of deformation nanotwins, which, compared to dislocations and shear bands, exhibit a higher resistance to thermal recovery.

This behavior is unambiguously confirmed by TEM observations and the corresponding SAED diffraction patterns (Figure 17a), which demonstrate the persistence of deformation nanotwins even after thermal exposure up to 500 °C. At 600 °C, a reduction in strength was observed for both processing routes (AR and CR), and with a further temperature increase up to 800 °C, partial recrystallization of the microstructure occurred [48], accompanied by the formation of annealing twins, as clearly reflected in the evolution of the mechanical properties shown in Figure 17b. Even within shear-band regions, deformation twins remained present after thermal treatment, indicating their pronounced microstructural stability (Figure 17c). This observation was further confirmed by TEM analyses and the corresponding SAED diffraction patterns, which documented the preservation of twin boundaries even in highly deformed regions. The presence of deformation twins within shear bands suggests that these defects contribute not only to the initial strain hardening but also to the stabilization of the microstructure during subsequent thermal loading.

## 5. Conclusions

In this work, the effect of multi-stage thermomechanical processing on the microstructural evolution and mechanical properties of the medium-entropy alloy Al_0.4_CrFe_2_Ni_2_ was analyzed. Based on the obtained results, the following conclusions can be drawn:

- The homogenization of the as-cast microstructure of the investigated MEA alloy through homogenization annealing followed by 80% hot rolling resulted in a chemically homogeneous, fine-grained polyhedral FCC microstructure with an increased fraction of annealing twins and a single-phase supersaturated solid solution, leading to a more stable plastic flow behavior of the material.
- The microstructural evolution during rolling under both temperature conditions (AR and CR) was predominantly governed by shear band formation; however, under cryogenic conditions (CR), a more pronounced deformation localization was observed, leading to the formation of deformation nanotwins and significantly enhanced strengthening. As a result, the CR60 condition achieved a yield strength of 1040 MPa and an ultimate tensile strength of 1235 MPa compared to 838 MPa (YS) and 1045 MPa (UTS) for the AR60 condition.
- Precipitation hardening of the alloy was achieved through the formation of B2 precipitates arranged in chain-like configurations along the shear bands, which significantly contributed to the enhancement of strength. The alloy processed under cryogenic conditions reached its maximum strengthening after annealing at 500 °C, exhibiting a yield strength of 1420 MPa and an ultimate tensile strength of 1465 MPa; this effect results from the synergistic contribution of a higher fraction of B2 precipitates and the presence of deformation nanotwins.
- The alloy exhibited good thermal stability up to 600 °C; however, upon exceeding this temperature and annealing at 800 °C, a pronounced deterioration of mechanical properties occurred, which was associated with partial recrystallization of the material.
- Overall, the investigated alloy offers a wide spectrum of tunable mechanical properties, which can be effectively tailored through appropriate combinations of thermomechanical processing routes, including rolling temperature, deformation degree, and subsequent heat treatment.

## Figures and Tables

**Table 1:** The chemical composition of the as-cast Al_0.4_CrFe_2_Ni_2_ alloy.

Region	Al	Cr	Fe	Ni
	at. %			
Nominal	7.1	18.52	37.04	37.04
Actual	8.99	17.11	36.25	37.65
Dendrite	8.84	17.98	36.99	36.19
Inter-dendrite	10.48	19.05	32.85	37.63

**Table 2:** Yield strength and ultimate tensile strength, total elongation to fracture, and reduction in area for the as-cast, homogenized, hot-rolled, ambient-rolled, and cryogenically rolled conditions.

State	YS [MPa] ± Dev	UTS [MPa] ± Dev	TE [%] ± Dev	RA [%] ± Dev
As-cast	268 ± 8	474 ± 2	44.9 ± 5	54.4 ± 5
HT	191 ± 6.5	403 ± 1.7	48.5 ± 3.1	59.9 ± 3.1
HR	288 ± 2	645 ± 1	70.1 ± 2.3	69.1 ± 1.1
AR20	738 ± 8.2	807 ± 6	21.0 ± 0.2	59.1 ± 0.4
AR40	812 ± 10	941 ± 6.5	12.1 ± 0.4	45.7 ± 1.5
AR60	839 ± 4.5	1045 ± 6	10.5 ± 0.3	41.3 ± 0.5
CR20	795 ± 7.5	868 ± 6.5	13.4 ± 1.2	51.6 ± 4.7
CR40	962 ± 9.9	1105 ± 5	9.4 ± 0.2	39.1 ± 0.5
CR60	1040 ± 9.2	1235 ± 5.6	6.4 ± 0.5	30.2 ± 0.2

**Table 3:** Lattice constants of Al_0.4_CrFe_2_Ni_2_ alloys after annealing.

Microstructure	Lattice Constant (Å)			
Samples	AR60-600	AR60-800	CR60-600	CR60-800
FCC	3.580	3.581	3.576	3.599
BCC	2.860	2.861	2.854	2.881

**Table 4:** The chemical composition of the Al_0.4_CrFe_2_Ni_2_ alloy after heat treatment.

Region	Al	Cr	Fe	Ni
	at. %			
Matrix	8.39	19.71	35.26	36.66
Fine precipitates	18.79	13.91	28.28	39.02
Coarse precipitates	27.63	8.55	18.01	45.82

**Table 5:** Mechanical property values (YS, UTS, engineering strain, and RA) after heat treatment of the AR60 and CR60 conditions.

	YS [MPa]	UTS [MPa]	ε [%]	RA [%]
AR60	839 ± 5	1045 ± 6	10.5 ± 0.3	41.3 ± 0.5
AR60-500	1110 ± 4.2	1253 ± 4.5	9.39 ± 1.5	21.8 ± 1.8
AR60-600	1130 ± 6.7	1320 ± 6.7	12.8 ± 0.6	26.3 ± 2.1
AR60-700	904 ± 8.3	1114 ± 7.5	16.8 ± 0.5	31.7 ± 1.5
AR60-900	613 ± 8.5	872 ± 9.1	21.7 ± 1.1	23.2 ± 1.7
CR60	1040 ± 10	1235 ± 5	6.4 ± 0.5	30.2 ± 0.2
CR60-400	1370 ± 6.8	1447 ± 4.3	4.91 ± 0.8	43.7 ± 2.8
CR60-500	1420 ± 5.5	1465 ± 5.1	5.5 ± 0.6	14.8 ± 1.3
CR60-600	1271 ± 9.3	1411 ± 7.3	8.4 ± 0.3	16.1 ± 4
CR60-700	911 ± 7.2	1024 ± 3.2	19.1 ± 0.9	19.0 ± 2.1
CR60-900	577 ± 11.2	859 ± 8.3	27.8 ± 0.7	29.2 ± 2

## Data Availability

The original contributions presented in this study are included in the article. Further inquiries can be directed to the corresponding author.

## References

[1] Lu W., Luo X., Ning D., Wang M., Yang C., Li M., Yang Y., Li P., Huang B. (2022). Excellent Strength-Ductility Synergy Properties of Gradient Nano-Grained Structural CrCoNi Medium-Entropy Alloy. J. Mater. Sci. Technol..

[2] Li Z., Pradeep K.G., Deng Y., Raabe D., Tasan C.C. (2016). Metastable High-Entropy Dual-Phase Alloys Overcome the Strength–Ductility Trade-Off. Nature.

[3] Chuang M.-H., Tsai M.-H., Wang W.-R., Lin S.-J., Yeh J.-W. (2011). Microstructure and Wear Behavior of AlxCo1.5CrFeNi1.5Tiy High-Entropy Alloys. Acta Mater..

[4] Lee W., Oh Y., Jo M.-G., Han H.N., Kim Y. (2021). Microstructures and Mechanical Properties of CrFeNi2Nbx Eutectic Multicomponent Alloys. J. Alloys Compd..

[5] Bała P., Górecki K., Dziurka R., Kozieł T. (2023). The Effect of Al and Ti Additions on Solid Solution Strengthening and Precipitation Hardening in CoNiFe Medium-Entropy Alloys. Materials.

[6] Wang W.-R., Wang W.-L., Wang S.-C., Tsai Y.-C., Lai C.-H., Yeh J.-W. (2012). Effects of Al Addition on the Microstructure and Mechanical Property of AlxCoCrFeNi High-Entropy Alloys. Intermetallics.

[7] Gwalani B., Gorsse S., Choudhuri D., Styles M., Zheng Y., Mishra R.S., Banerjee R. (2018). Modifying Transformation Pathways in High Entropy Alloys or Complex Concentrated Alloys via Thermo-Mechanical Processing. Acta Mater..

[8] Dasari S., Sarkar A., Sharma A., Gwalani B., Choudhuri D., Soni V., Manda S., Samajdar I., Banerjee R. (2021). Recovery of Cold-Worked Al0.3CoCrFeNi Complex Concentrated Alloy through Twinning Assisted B2 Precipitation. Acta Mater..

[9] Gwalani B., Soni V., Lee M., Mantri S., Ren Y., Banerjee R. (2017). Optimizing the Coupled Effects of Hall-Petch and Precipitation Strengthening in a Al0.3CoCrFeNi High Entropy Alloy. Mater. Des..

[10] Yasuda H.Y., Miyamoto H., Cho K., Nagase T. (2017). Formation of Ultrafine-Grained Microstructure in Al0.3CoCrFeNi High Entropy Alloys with Grain Boundary Precipitates. Mater. Lett..

[11] Annasamy M., Haghdadi N., Taylor A., Hodgson P., Fabijanic D. (2019). Static Recrystallization and Grain Growth Behaviour of Al0.3CoCrFeNi High Entropy Alloy. Mater. Sci. Eng. A.

[12] Wang X., Zhang Z., Wang Z., Ren X. (2022). Excellent Tensile Property and Its Mechanism in Al0.3CoCrFeNi High-Entropy Alloy via Thermo-Mechanical Treatment. J. Alloys Compd..

[13] Mahata C., Kumar K.N., Bruder E., Gowtam D., Sadhasivam M., Durst K., Pradeep K.G. (2025). Recovery of Metastable Solid Solution from a Severe Plastically Deformed Cu Alloyed FeMnCoCr High Entropy Alloy. J. Alloys Metall. Syst..

[14] Yoshida S., Park N., Shiotani K., Bai Y., Niiyama T., Shibata A., Shimokawa T., Tsuji N. (2025). Ultra-Grain-Refinement of Face-Centered Cubic High/Medium-Entropy Alloys: Approaching the Limit of Grain Refinement through Severe Plastic Deformation and Recrystallization. Mater. Des..

[15] Ren Q., Li T., Xie H., Jia Y., Wan M., Huang C., Chen C., Li J., Lu Y. (2025). Achieving Synergistic Strength-Ductility Enhancement in a Hierarchical Hetero-Lamellar AlCoCrFeNi2.1 Eutectic High-Entropy Alloy via Facile Hot-Rolling Strategy. Mater. Des..

[16] Kumar P., Jain V., Samal S., Ghosh A. (2024). Effect of Hot Rolling and Annealing on Microstructure, Crystallographic Texture, and Mechanical Properties of Fe11.5 Co20.6 Ni40.7 Cr12.2 Al7.8 Ti7.2 High Entropy Alloy. Mater. Sci. Eng. A.

[17] Liao Y., Song Y., Shu N., Niu Y., Zhang H., Sun B., Wang Y., Li C., Gu J. (2025). Enhanced Strength-Ductility Synergy in Ferrous Medium-Entropy Alloys via Single-Step Hot Rolling. Mater. Sci. Eng. A.

[18] Liu M., Zhang S., Li F., Luo Y., Yao Y., Zhang H., Wang Z., Wang L., Wang Z. (2021). Tailoring the Strength and Ductility of Al0.25CoCrFeNi High Entropy Alloy through Cryo-Rolling and Annealing. Mater. Sci. Eng. A.

[19] Zhang X., Dong R., Guo Q., Hou H., Zhao Y. (2023). Predicting the Stacking Fault Energy in FCC High-Entropy Alloys Based on Data-Driven Machine Learning. J. Mater. Res. Technol..

[20] Xiao Q., Wang L., Liang Y.-J., Xue Y. (2021). Annealing Hardening in Cryo-Rolled High-Entropy Alloys by Belated Deformation Twinning. Mater. Sci. Eng. A.

[21] Deng Y., Tasan C.C., Pradeep K.G., Springer H., Kostka A., Raabe D. (2015). Design of a Twinning-Induced Plasticity High Entropy Alloy. Acta Mater..

[22] Lilensten L., Couzinié J.-P., Bourgon J., Perrière L., Dirras G., Prima F., Guillot I. (2017). Design and Tensile Properties of a Bcc Ti-Rich High-Entropy Alloy with Transformation-Induced Plasticity. Mater. Res. Lett..

[23] Tirunilai A.S., Sas J., Weiss K.-P., Chen H., Szabó D.V., Schlabach S., Haas S., Geissler D., Freudenberger J., Heilmaier M. (2018). Peculiarities of Deformation of CoCrFeMnNi at Cryogenic Temperatures. J. Mater. Res..

[24] Sun X., Zhang H., Li W., Ding X., Wang Y., Vitos L. (2020). Generalized Stacking Fault Energy of Al-Doped CrMnFeCoNi High-Entropy Alloy. Nanomaterials.

[25] Liu S.F., Wu Y., Wang H.T., Lin W.T., Shang Y.Y., Liu J.B., An K., Liu X.J., Wang H., Lu Z.P. (2019). Transformation-Reinforced High-Entropy Alloys with Superior Mechanical Properties via Tailoring Stacking Fault Energy. J. Alloys Compd..

[26] Zhao Y., Chen Z., Yan K., Le W., Naseem S., Zhang H., Yang L. (2023). Investigation on Microstructure, Superior Tensile Property and Its Mechanism in Al0.3CoCrFeNi High-Entropy Alloy via Thermo-Mechanical Processing. Mater. Sci. Eng. A.

[27] Liu S., Gao H., Wei D., Kong C., Kumara L.S.R., Fu M.W., Yu H. (2024). Deformation Mechanism of a Metastable Medium Entropy Alloy Strengthened by the Synergy of Heterostructure Design and Cryo-Pre-Straining. Int. J. Plast..

[28] Xu X., Li H., Zhao Y., Zhang X., Pan Y., Liaw P.K., Hou H. (2025). Enhancement of Strength-Ductility Trade-off in Al5Fe25Cr25Ni42.5Ti2.5 High Entropy Alloy through Annealing Twins. Mater. Sci. Eng. A.

[29] Xu X., Song Z., Wang K., Li H., Pan Y., Hou H., Zhao Y. (2025). Cryo-Rolling and Annealing-Mediated Phase Transformation in Al5Ti2.5Fe25Cr25Ni42.5 High-Entropy Alloy: Experimental, Phase-Field and CALPHAD Investigation. J. Mater. Sci. Technol..

[30] Sang P., Liang N., Liu Y., Gu L., Zhang Z., Niu K., Wang S., Yang X.-S., Li Y. (2025). Synergistic Improvement of Strength and Ductility by Nano-Lamellar L12 Precipitates in Co-Free NiFeCrAlTi Medium-Entropy Alloy. Mater. Sci. Eng. A.

[31] Kočiško R., Petroušek P., Saksl K., Petryshynets I., Milkovič O., Csík D. (2025). The Influence of Ti and Al on the Evolution of Microstructure and Mechanical Properties in Medium-Entropy and High-Entropy Alloys Based on AlxTixCrFe2Ni2. Materials.

[32] Hammersley A.P., Svensson S.O., Hanfland M., Fitch A.N., Hausermann D. (1996). Two-Dimensional Detector Software: From Real Detector to Idealised Image or Two-Theta Scan. High Press. Res..

[33] (2022). Metallic Materials—Tensile Testing—Part 1: Method of Test at Room Temperature.

[34] (2023). Metallic Materials—Vickers Hardness Test.

[35] Tripathy B., Malladi S.R.K., Bhattacharjee P.P. (2022). Development of Ultrafine Grained Cobalt-Free AlCrFe2Ni2 High Entropy Alloy with Superior Mechanical Properties by Thermo-Mechanical Processing. Mater. Sci. Eng. A.

[36] Zhou J., Liao H., Chen H., Feng D., Zhu W. (2024). Effect of Cold Rolling on Microstructure and Mechanical Behavior of Fe35Ni35Cr20Mn10 High-Entropy Alloy. Mater. Charact..

[37] Wang W., Zhou W., Song S., Reddy K.M., Wang X. (2020). Effect of Deformation Induced B2 Precipitates on the Microstructure and Mechanical Property of Al0.3CoCrFeNi High-Entropy Alloy. J. Alloys Compd..

[38] Liao Y.C., Chen P.S., Tsai P.H., Jang J.S.C., Hsieh K.C., Chang H.W., Chen C.Y., Huang J.C., Wu H.J., Lo Y.C. (2022). Effect of Thermomechanical Treatment on the Microstructure Evolution and Mechanical Properties of Lightweight Ti65(AlCrNb)35 Medium-Entropy Alloy. Intermetallics.

[39] Moemeni S., Dehghani K. (2025). Exploring the Coupled Effect of Cold Rolling Level and Annealing Parameters on the Development of Microstructure, Mechanical Properties and Texture of Al0.3CoCrFeNi High Entropy Alloy. Mater. Today Commun..

[40] Gwalani B., Gorsse S., Choudhuri D., Zheng Y., Mishra R.S., Banerjee R. (2019). Tensile Yield Strength of a Single Bulk Al0.3CoCrFeNi High Entropy Alloy Can Be Tuned from 160 MPa to 1800 MPa. Scr. Mater..

[41] Sathiyamoorthi P., Asghari-Rad P., Park J.M., Moon J., Bae J.W., Zargaran A., Kim H.S. (2019). Exceptional Cryogenic Strength-Ductility Synergy in Al0.3CoCrNi Medium-Entropy Alloy through Heterogeneous Grain Structure and Nano-Scale Precipitates. Mater. Sci. Eng. A.

[42] Microstructure and Tensile Behaviors of FCC Al0.3CoCrFeNi High Entropy Alloy—ScienceDirect. https://www.sciencedirect.com/science/article/pii/S0925838808022780#fig2.

[43] Duan T., Li J., Zou G., Chen S., Ye L. (2025). Effect of Deformation-Induced Precipitation of B2 Phase on Superplasticity of Al0.5CoCrFeNiCu High-Entropy Alloy. Mater. Lett..

[44] Li Z., Wang L., Liu C., Zhao J., Wang B., Li Z., Luo L., Chen R., Su Y., Guo J. (2023). Hall-Petch Strengthening by Grain Boundaries and Annealing Twin Boundaries in Non-Equiatomic Ni_2_FeCr Medium-Entropy Alloy. Metals.

[45] Chen P.-S., Shiu S.-J., Tsai P.-H., Liao Y.-C., Jang J.S.-C., Wu H.-J., Chang S.-Y., Chen C.-Y., Tsao I.-Y. (2022). Remarkable Enhanced Mechanical Properties of TiAlCrNbV Medium-Entropy Alloy with Zr Additions. Materials.

[46] Gonçalves C.N., Paul M.J., Webster R.F., Kong C., Gludovatz B., Zepon G., Coury F.G., Mazzer E.M. (2024). Impact of Rolling Temperature on the Deformation Structure and Mechanical Performance of a CrMnFeCoNi High-Entropy Alloy. J. Alloys Compd..

[47] Lv Y., Wang Y., Zhang J., Lei Y., Song P., Ding R., Yao X., Chen J. (2024). A Novel High Entropy Alloy with Outstanding Strength by Low Temperature Annealing after Severe Cold Rolling. J. Mater. Res. Technol..

[48] Zhang Z., Cheng Y., Wang X., Song S., Ren X. (2025). Investigation on Tensile Property and Mechanism of Partially Recrystallized Al0.1CoCrFeNi High-Entropy Alloy after Cold Rolling and Annealing Treatment. Mater. Sci. Eng. A.

[49] Liu X.X., Ma S.G., Song W.D., Zhao D., Wang Z.H. (2023). Microstructure Evolution and Mechanical Response of Co-Free Ni2CrFeAl0.3Tix High-Entropy Alloys. J. Alloys Compd..

[50] Fedoriková A., Petroušek P., Kvačkaj T., Kočiško R., Zemko M. (2023). Development of Mechanical Properties of Stainless Steel 316LN-IG after Cryo-Plastic Deformation. Materials.

[51] Stepanov N., Tikhonovsky M., Yurchenko N., Zyabkin D., Klimova M., Zherebtsov S., Efimov A., Salishchev G. (2015). Effect of Cryo-Deformation on Structure and Properties of CoCrFeNiMn High-Entropy Alloy. Intermetallics.

